# 3D finite-element brain modeling of lateral ventricular wall loading to rationalize periventricular white matter hyperintensity locations

**DOI:** 10.1007/s00366-022-01700-y

**Published:** 2022-07-19

**Authors:** Andreia Caçoilo, Henry Rusinek, Johannes Weickenmeier

**Affiliations:** 1 Department of Mechanical Engineering, Stevens Institute of Technology, Hoboken, NJ 07030, USA; 2 Department of Radiology, New York University Grossman School of Medicine, New York, NY 10016, USA

**Keywords:** Periventricular white matter hyperintensities, Ventricular wall loading, Ependymal cell stretch, Computational modeling, Personalized finite-element simulations

## Abstract

Aging-related periventricular white matter hyperintensities (pvWMHs) are a common observation in medical images of the aging brain. The underlying tissue damage is part of the complex pathophysiology associated with age-related microstructural changes and cognitive decline. PvWMH formation is linked to blood–brain barrier dysfunction from cerebral small vessel disease as well as the accumulation of cerebrospinal fluid in periventricular tissue due to progressive denudation of the ventricular wall. In need of a unifying theory for pvWMH etiology, image-based finite-element modeling is used to demonstrate that ventricular expansion from age-related cerebral atrophy and hemodynamic loading leads to maximum mechanical loading of the ventricular wall in the same locations that show pvWMHs. Ventricular inflation, induced via pressurization of the ventricular wall, creates significant ventricular wall stretch and stress on the ependymal cells lining the wall, that are linked to cerebrospinal fluid leaking from the lateral ventricles into periventricular white matter tissue. Eight anatomically accurate 3D brain models of cognitively healthy subjects with a wide range of ventricular shapes are created. For all models, our simulations show that mechanomarkers of mechanical wall loading are consistently highest in pvWMHs locations (*p* < 0.05). Maximum principal strain, the ependymal cell thinning ratio, and wall curvature are on average 14%, 8%, and 24% higher in pvWMH regions compared to the remaining ventricular wall, respectively. Computational modeling provides a powerful framework to systematically study pvWMH formation and growth with the goal to develop pharmacological interventions in the future.

## 1 Introduction

White matter lesions are a common feature of nearly every aging brain [1, 2]. On fluid-attenuated inversion recovery-based magnetic resonance images, or simply FLAIR, these lesions show as bright-appearing spots typically observed in either subcortical or periventricular white matter regions [3, 4]. Clinically, white matter hyperintensities (WMHs) strongly correlate with aging and cardiovascular risk factors including smoking, diabetes, and hypertension [5, 6]. They are linked to cognitive decline in the form of reduced memory and motor function, and are associated with an increased risk for stroke [7–11]. WMHs are also an accompanying pathology in normal pressure hydrocephalus [12], multiple sclerosis [13, 14], and neurodegenerative diseases such as Alzheimer’s and Parkinson’s disease [1, 15]. Several newly available in vivo MRI methods inform us about microstructure (including anisotropy, diffusivity, and fluid composition) of WMH pathologies [16–18]. These studies increasingly demonstrate that white matter undergoes structural damage prior to transitioning to lesions categorized as WMHs [19–22]. Imaging, however, is inherently limited to discrete snapshots of a subject’s current state of health and sparse longitudinal monitoring of WMH initiation and progression [23, 24]. The increasing availability of longitudinal data, i.e., multiple scans of the same subjects across a time-span of several years, is providing increasing evidence that WMHs co-evolve with a series of age-related changes, including cerebral atrophy, small vessel disease, and ventricular enlargement [25, 26].

The differentiation between deep WMHs (dWMHs) and periventricular WMHs (pvWMHs) is part of an ongoing debate about the need for separate pathophysiological mechanisms underlying their respective initiation and progression [27–32]. PvWMHs consistently appear in the anterior and posterior horns and along the edges of the main body, while dWMHs emerge in diffuse subcortical white matter regions and do not coincide with any particular anatomical feature [4, 33]. From a mechanics perspective, the periventricular region is subject to increased tissue loading in comparison to other subcortical regions due to the shape of the lateral ventricles and the pulsatile expansion of the ventricles from hemodynamic loads [34–36]. Previous work showed that mechanical loads along the ventricular wall peak in the ventricular horns and demonstrated a statistically significant spatial overlap of maximum wall stretch and pvWMH locations on the basis of eight subjects with various lateral ventricular shapes [36]. Today, the most widely accepted mechanism for pvWMHs initiation is associated with cerebral small vessel disease and the progressive dysfunction of the blood–brain barrier leading to an accumulation of proteins and metabolites in white matter tissue. This toxic buildup drives demyelination and neuroinflammation [6]. Blood–brain barrier dysfunction is typically supported by diffusion tensor imaging that shows noticeable changes in water content, loss of fractional anisotropy, and higher mean diffusivity, all of which are indicators for pronounced white matter degeneration [18, 22, 31].

In comparison to the many imaging-based studies on WMHs, only few studies investigated the pathological changes associated with aging of the ventricular wall and periventricular tissues, e.g., [37–40]. Common observations include denudation of the ventricular wall and astrogliosis, accumulation of fluid in periventricular tissue due to cerebrospinal fluid (CSF) leakage, and white matter demyelination leading to axon damage [38, 41, 42]. Locations of elevated mean curvature, i.e., the horns and the edges of the main body, are reported to show increased signs of ependymal cell loss and markers for demyelination and neuroinflammation [39, 43].

**Fig. 1:**
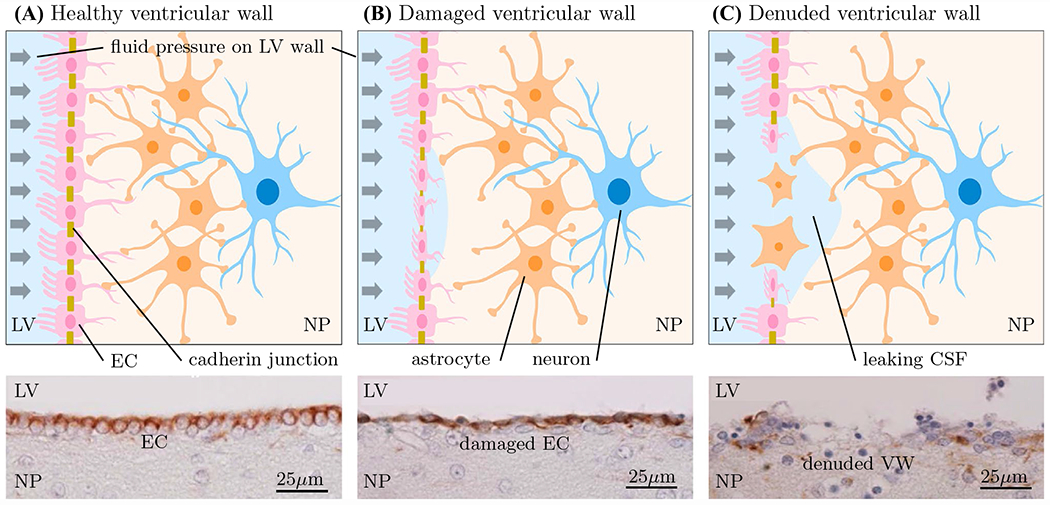
Top row: schematic of ventricular wall degeneration. **A** The intact ependyma consists of ciliated ependymal cells connected through tight cadherin junctions. Fluid pressure caused by every heartbeat leads to oscillatory inflation of the ventricular wall which stresses ependymal cells and intercellular junctions. **B** This continuous ependymal cell stretch causes CSF to leak into the parenchyma due to stressed cadherin junctions. **C** Gradual loss of ependymal cells leads to astrogliosis and dysfunctional fluid exchange between the lateral ventricles and parenchyma. In turn, fluid diffuses into deep white matter tissue and drives lesion formation and growth. Bottom row: histological images adapted from [44]. *LV* lateral ventricles, *EC* ependymal cells, *NP* neuropil, *VW* ventricular wall

Figure 1 shows a schematic of the damage mechanism underlying the present work. Figure 1A shows the healthy ventricular wall that is lined with ciliated cuboidal ependymal cells which provide an immunological barrier and semi-permeable membrane for bi-directional transport of molecules between CSF and parenchyma [45]. Ependymal cells lateral sides are populated with cadherin junctions that form tight connections between individual ependymal cells as well as specialized transporters that enable selective transport of molecules [45]. Hemodynamic pulsation and cerebral atrophy lead to a loading state of the ventricular wall, see Fig. 1B, that causes ependymal cells to be stretched thin. Subsequent leakage of CSF into deep white matter tissue first forms the pencil thin linings observed in early stages of pvWMHs. Over time, additional fluid accumulates and diffuses into deeper tissue layers to form lesion caps, and ultimately reaches deep white matter tissue [3, 24, 26], see Fig. 1C. The locally degrading ependymal cell layer is replaced by astrogliosis. The accumulation of excess fluid in the presence of age-related cerebral small vessel disease and dysfunction of the blood–brain barrier gives rise to accelerated pvWMH growth as time progresses [19, 23, 24].

In previous work, we presented a modeling approach that predicts possible pvWMH locations on the basis of subject-specific 2D models [36]. From the imaging database of the New York University Alzheimer’s Disease Research Center, we selected eight cognitively normal subjects with a wide range of ventricular volumes. Based on structural MRI from each subject, we identified the axial slice with the largest lateral ventricular area, segmented gray matter, white matter, and fluid spaces, and converted these regions of interest into a finite-element (FE) model. To approximate the effect of the brain’s pulsatile loading during every heartbeat we performed a quasi-static simulation and prescribed a normal pressure on the ventricular wall and the CSF–gray matter interface. We demonstrated that peak ependymal cell stretch localizes in the anterior and posterior horns of the lateral ventricles and observed statistically significant overlap between pvWMH locations and maximum cell stretch. In the present work, we extend the 2D modeling framework to full 3D finite-element simulations and introduce additional mechanomarkers to rationalize the correlation between ventricular wall loading and onset locations of pvWMHs on the ventricular surface. The extension to 3D simulations improves the prediction of ventricular wall loading and further substantiates our claim that ependymal cell stretch drives pvWMH formation. It also represents the next step towards developing a multiphysics model to simulate long-term pvWMH changes, i.e., the progressive growth of periventricular white matter lesions into deep white matter regions.

## 2 Methods

### 2.1 Subject selection and imaging acquisition

**Fig. 2:**
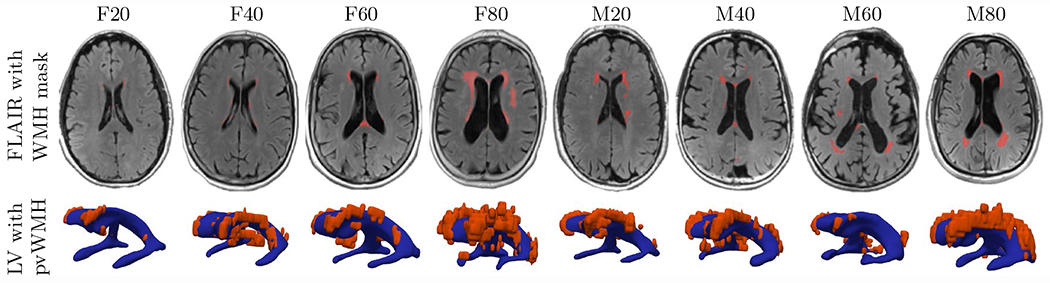
Representative axial FLAIR images for each of the eight different subjects with a white matter hyperintensity (WMH) mask (in red) in the first row, and the corresponding reconstruction of the lateral ventricles (LV) (in blue) and the pvWMHs (in red) in the second row. F20 through F80 and M20 through M80 stand from female/male subjects from the 20th through the 80th percentile with respect to total CSF volume (color figure online)

Magnetic resonance images (MRI) were obtained from the imaging database of the New York University Alzheimer’s Disease Research Center. Each participant provided institutional review board approved consent for a protocol investigating risk factors of cognitive decline and Alzheimer’s disease. Cognition was evaluated via the Brief Cognitive Rating Scale and Global Deterioration Scale (GDS) [46]. Subjects with tumors, neocortical infarctions, multiple sclerosis, and diabetes were excluded; additionally, subjects using psychoactive medications and scoring < 16 on the 17-item Hamilton Depression Scale were also excluded [47]. Only subjects who had scored at least 27 points on the Mini Mental State Examination [48] and scored a GDS = 1, i.e., no subjective memory complaints, or GDS = 2, i.e., with subjective memory complaints, but not fulfilling the criteria for mild cognitive impairment or dementia, were included. These inclusion criteria yielded a subset of *N* = 352 cognitively healthy elderly: 209 women, 68.1 ± 8.0 years old (mean ± standard deviation) and 143 men, age 71.8 ± 7.3. Each subject underwent structural MRI on a 3T Siemens Magnetom Prisma (Siemens Healthineers USA). The exam included a high-resolution T1-weighted MPRAGE sequence (TR = 2100 ms, TE = 5 ms, TI = 900 ms, FA = 9°, 256 × 256 × 176 matrix, 1 × 1 × 1 mm voxels, GRAPPA2 acceleration) and a FLAIR sequence used to assess white matter lesions (TR = 9000 ms, TE = 75 ms, TI = 2500 ms, FA = 120°, 320 × 196 × 40, 0.7 × 0.7 × 4 mm voxels, GRAPPA2 acceleration). Gray matter, white matter, and CSF were segmented on MPRAGE images using Statistical Parametric Mapping Version 12 implemented in Matlab [49]. Brain aging is associated with cerebral atrophy and ventricular enlargement. Since age may significantly differ from biological age, i.e. amount of atrophy, all subjects were sorted by gender and based on their total intracranial CSF volume [50]. To capture a broad range of ventricular geometries, male and female subjects from the 20th, 40th, 60th, and 80th percentile of total intracranial CSF volume were selected. Subjects were labeled as F20/F40/F60/F80 (females) and M20/M40/M60/M80 (males), respectively, and representative axial slices are shown in Fig. 2 (top row). By selecting subjects from a wide range of CSF volumes, our study indirectly incorporated (biological) age. Subjects’ Fazekas scores were assessed by two neurologists from the New York Langone Medical Center. CSF and lateral ventricular volume were derived from Freesurfer segmentations of the MPRAGE images. For all eight subjects, WMHs were segmented on FLAIR images with FireVoxel (build 301, www.firevoxel.org), see Fig. 2 (bottom row). The automatic WMH segmentation algorithm first performs uniformity correction [51] and then estimates signal intensity within an image-dependent whole-brain mask Ω. The WMHs were then segmented by thresholding from Ω all voxels *v*, such that $\bar{ℳ}=\left\{v|v\in \text{Ω}\wedge \left(v\right)>\mu +k\cdot \sigma \right\}$, where s(v) is the intensity of voxel *v*, *μ* is the mean intensity value, *σ* is the standard deviation of intensity distribution in Ω, and *k* was set at 2.5. In a last step, septum and choroid plexus are deleted from $\bar{ℳ}$. These structures are identified as connected components of $\bar{ℳ}$ having > 50% surface boundary adjacent to CSF. The resulting WMH masks ℳ were verified by neuroscientists from the New York University Alzheimer’s Disease Research Center.

### 2.2 MRI segmentation and finite-element model generation

#### 2.2.1 Brain and pvWMH segmentation

**Fig. 3:**
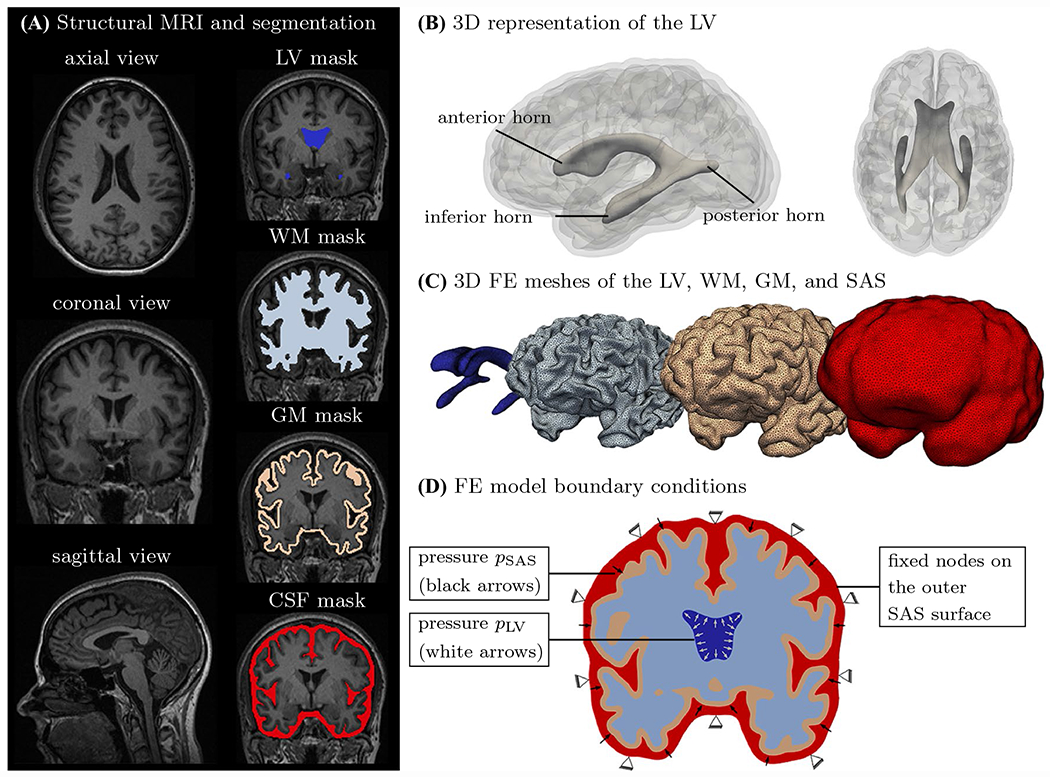
Semi-automatic image-based FE brain model generation. **A** Representative axial, coronal, and sagittal view of the brain. The models differentiate between lateral ventricle (LV), white matter (WM), gray matter (GM), and CSF. **B** 3D reconstruction of the LV with labels identifying the anterior, posterior, and inferior horns. **C** Visualization of a representative 3D brain model. **D** Zero-displacement boundary conditions are prescribed to the outer CSF surface to fix the model in space. Pressure from circulating CSF on parenchyma is modeled via a normal pressure on the CSF–gray matter interface, *p*_SAS_ (SAS = subarachnoid space); lateral ventricular expansion at peak hemodynamic load is modeled as a normal pressure on the LV–white matter interface, *p*_LV_

Eight subject-specific, anatomically accurate, three-dimensional finite-element brain models were created based on a previously developed semi-automatic segmentation approach [52]. Each subject’s MPRAGE scan was imported into the “ScanIP” module of the commercial software Simpleware (Synopsis Inc, Mountain View CA) for segmentation and finite-element mesh generation. Gray-scale thresholding and manual correction was used to delineate the lateral ventricles, white matter, gray matter, and the subarachnoid space, as shown in Fig. 3. The binarized WMH masks ℳ were also imported into ScanIP to reconstruct pvWMH volumes. Segmentation volumes were converted into a FE mesh consisting of linear tetrahedral elements using the “FE Model” module. Minimum and maximum element edge length was set to 1.0–1.2 mm for the lateral ventricles and pvWMHs, and 2.0–2.5 mm for white matter, gray matter, and subarachnoid space.

#### 2.2.2 Constitutive material model

Brain tissue is assumed to behave like a purely hyperelastic material [52]. Following the continuum theory of finite deformations, the deformation gradient **F** is defined as the gradient of the nonlinear deformation field *ϕ* with respect to material coordinates **X** in the reference configuration. Assuming that brain tissue behaves nearly incompressible, the deformation gradient **F** is split into a volumetric contribution characterized through the Jacobian *J* and an isochoric contribution $\bar{F}$,

(1) $$\text{F}=\nabla_{\text{X}}\phi =J^{1/3}\bar{\text{F}},\;\text{with}\;J=\det\left(\text{F}\right)\;\text{and}\;\bar{\text{F}}=J^{-1/3}\text{F}.$$

As a characteristic deformation measure, the right Cauchy–Green deformation tensor **C** is introduced. **C** obeys a similar decomposition into a volumetric contribution in terms of the Jacobian *J* and an isochoric contribution $\bar{C}$,

(2) $$\text{C}={\text{F}}^{T}\cdot \text{F}=J^{2/3}\bar{\text{C}},\;\text{with}\;J^{2/3}=\det^{2/3}\left(\text{F}\right)\;\text{and}\;\bar{\text{C}}={\bar{\text{F}}}^{T}\bar{\text{F}}.$$

From **C**, the isochoric first and second invariants, ${\bar{I}}_{1}$ and ${\bar{I}}_{2}$, can be derived in terms of the isochoric right Cauchy–Green deformation tensor $\bar{C}$, or in terms of the isochoric principal stretches ${\bar{\lambda }}_{1},{\bar{\lambda }}_{2}$, and ${\bar{\lambda }}_{3}$, recalling that ${\bar{I}}_{3}=J^{2}=1$,

(3) $${\bar{I}}_{1}=\text{tr}\left(\bar{\text{C}}\right)={\bar{\lambda }}_{1}^{2}+{\bar{\lambda }}_{2}^{2}+{\bar{\lambda }}_{3}^{2},\;{\bar{I}}_{2}=\frac{1}{2}\left[{\text{tr}}^{2}\left(\bar{\text{C}}\right)-\text{tr}\left({\bar{\text{C}}}^{2}\right)\right]={\bar{\lambda }}_{1}^{2}{\bar{\lambda }}_{2}^{2}+{\bar{\lambda }}_{2}^{2}{\bar{\lambda }}_{3}^{2}+{\bar{\lambda }}_{3}^{2}{\bar{\lambda }}_{1}^{2}.$$

It has been shown that the quasi-static mechanical response of brain tissue is well captured by a one-term Ogden model given by the strain energy density function, **Ψ**, [36, 53],

(4) $$\Psi =\frac{\mu }{2}\left[{\bar{\lambda }}_{1}^{2}+{\bar{\lambda }}_{2}^{2}+{\bar{\lambda }}_{3}^{2}-3\right]+\frac{\kappa }{4}\left[J^{2}-1-2\;\log\left(J\right)\right],$$

with shear modulus μ governing isochoric, distortional deformations and bulk modulus κ governing dilatational deformations [54, 55]. White and gray matter are assumed to be nearly incompressible with a Poisson’s ratio of 0.45 and a white-to-gray matter stiffness ratio of two [56]. Specifically, experimentally-informed constants of μ=0.34 kPa and κ=3.3 kPa for gray matter and μ=0.68 kPa and κ=6.6 kPa for white matter were chosen [56–59]. For simulations conducted here, lateral ventricle elements were removed from the models and the subarachnoid space was modeled as an ultrasoft compressible material with a Young’s modulus of 1.0 Pa and a Poisson’s ratio of 0.30 [60].

#### 2.2.3 Numerical implementation in Abaqus

**Fig. 4:**
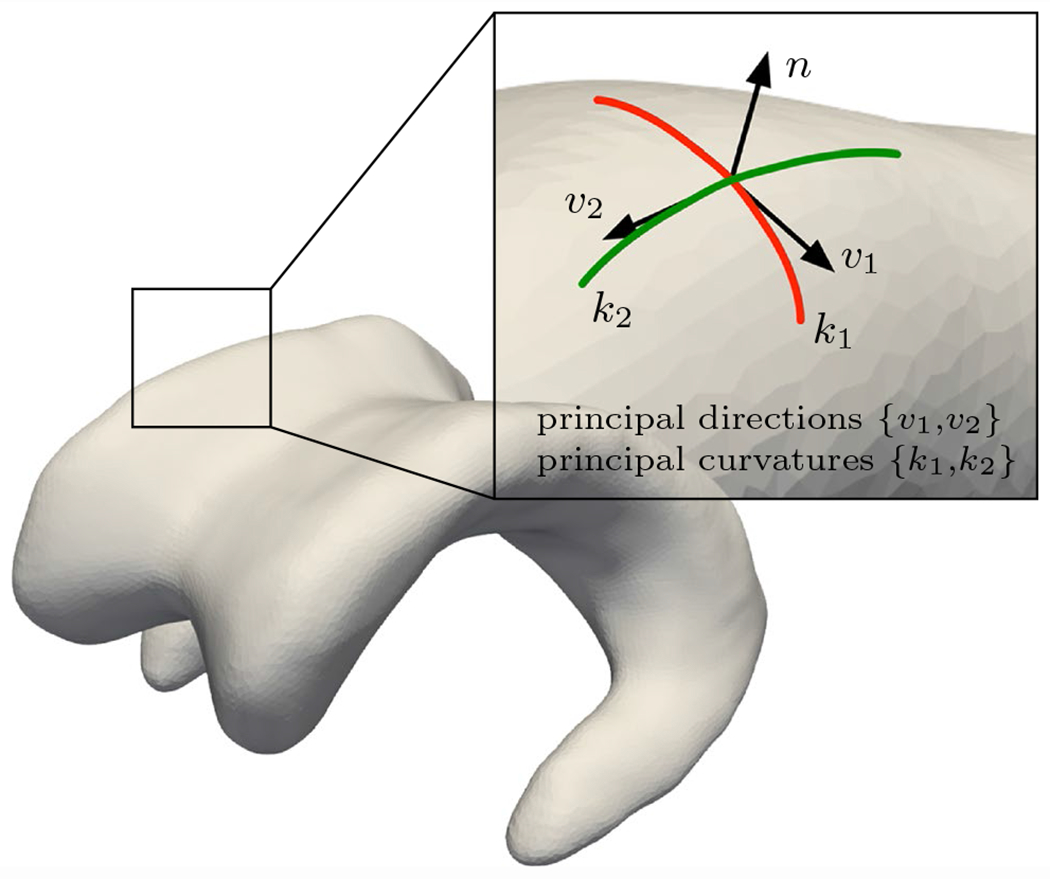
Curvature representation at a given location of a 3D lateral ventricle surface. The principal directions *v*_1_ and *v*_2_ form an orthonormal basis with the corresponding normal vector on the surface, while *k*_1_ and *k*_2_ are the principal curvatures

All numerical simulations were conducted using the commercial finite-element code Abaqus (Dassault Systémes, Providence, RI). To that end, the Ogden model was implemented in a user material subroutine (UMAT) following the example of Connolly et al. [61]. Ependymal cell loading is evaluated on the basis of characteristic stretches calculated from the projection of the right Cauchy Green tensor **C**,

(5) $$\lambda_{i}=\sqrt{{\text{r}}_{\text{i}}\cdot \text{C}\;{\text{r}}_{\text{i}}},\;\text{with}\;i=1,2,3,$$

where **r**_*i*_ are three orthonormal directions obtained from a diffusion problem on each subject’s model a priori. For the elements forming the ventricular wall, the objective is to determine the vectors that are normal to the ventricular surface as well as the two corresponding directions tangential to the wall for which the tangential stretch value is the highest and lowest, respectively. Solving the Laplacian diffusion problem with fixed temperature boundary conditions of *T* = 1 for all nodes on the ventricular surface, *T* = 0.1 for all nodes forming the gray matter–CSF interface, and *T* = 0 for all nodes on the outer surface of the model, provides the gradient field, i.e., heat flux, that corresponds to the desired directions. At every integration point, the normal stretch $\lambda_{n}$, maximum tangential stretch $\lambda_{t}^{\max}$, and minimum tangential stretch $\lambda_{t}^{\min}$ are calculated and stored as user-defined field variables in subsequent wall loading simulations. See Fig. 4 for clarification with regards to the projection directions $\left\{n,v_{1},v_{2}\right\}$ along the ventricular wall.

Ependymal cell loading simulations are treated as a quasi-static simulation to peak hemodynamic pressure during a single heartbeat. Each subject’s model is fixed in space via zero-displacement boundary conditions applied to all nodes on the outer surface of the model; this allows to omit the skull as an additional substructure in the model. Peak hemodynamic loading is represented by a surface pressure load that mimics the load of the pulsating fluid exerted onto the parenchyma. More specifically, a pressure of 300 Pa is applied to the ventricular surface acting against white matter as well as a pressure of 290 Pa is applied to the gray matter–CSF interface acting against gray matter. These values are based on measurements of pressure changes during the cardiac cycle derived from intracranial pressure waveforms in humans [62]. A peak-to-peak pressure amplitude of 2.3 mmHg, or ~ 300 Pa, was reported. The difference between lateral ventricular and CSF pressure is based on experiments on dog brains that revealed that a pressure difference as low as 5–10 Pa was sufficient to circulate CSF within lateral ventricles and the subarachnoid space [63, 64].

### 2.3 Mechanomarkers of ventricular wall loading

Ventricular wall loading is quantified on the basis of two mechanomarkers specifically developed to suit the morphology and anatomy of the lateral ventricles. These mechanomarkers quantify cell deformation and ventricular geometry.

#### 2.3.1 Ependymal cell thinning

The cyclic inflation of the lateral ventricles produces a deformation pattern that subjects cuboidal ependymal cells to repeated planar stretch while undergoing compression in the apical-basal axis [65]. To compare cell stretch patterns across multiple subjects, a thinning ratio, 𝒯, is defined as $𝒯=\left(\lambda_{t}^{\max},\lambda_{t}^{\min}\right)/\lambda_{n}$, i.e., area change divided by thickness change. In the literature, ependymal cells are described as being “stretched thin” in locations where the ventricular wall is damaged [43–45]. These observations are predominantly based on histological images that provide a 2D representation of what ependymal cells experience, see Fig. 1. In reality, however, ependymal cells undergo a three-dimensional loading state. Ependymal cells are not only compressed (thinning in 2D) but are also stretched tangentially to the ventricular wall which is similar to a biaxial loading state. Tangential stretch is much more relevant for pvWMH formation because it creates significant tension on intercellular adherence junctions leading to fluid leakage from the lateral ventricles into periventricular tissues. The proposed thinning ratio reflects ependymal cell’s 3D loading state.

#### 2.3.2 Mean ventricular curvature

Understanding changes of lateral ventricular geometry helps appreciate pvWMH formation as a longer term process that emerges in younger brains (with sharp horns) and grows as the brain atrophies. Moreover, clinicians have a better intuition for shapes and geometry (of the lateral ventricle and its horns, for example) compared to measures of mechanical loading (such as strain and stretch). Therefore, we introduce mean curvature 𝒦 to demonstrate the spatial overlap between regions of high curvature with pvWMH locations. Mean curvature reveals locations of the ventricular wall that are highly curved and, therefore, expose ependymal cells to increased mechanical loading. The curvature of the ventricular surface was calculated based on a method developed by Sacks et al. [66]. For each node of the ventricular surface mesh, a bi-quadratic surface patch is fitted to the nodes of neighboring elements. The surfaces are constructed from unit vectors $e_{u},e_{v}$ and $e_{n}$, where directions *u* and *v* are tangential to the surface and *n* is the normal direction to the surface. The resulting patch is represented by

(6) $$S\left(u,v\right)=a\;u^{2}+2b\;u\;v+c\;v^{2}+d\;u+e\;v+f,$$

where {a,b,c,d,e,f} are fitting constants determined for each surface patch. The ventricular surface may be considered smooth such that constant *f* can be eliminated from Eq. (6); moreover, since the computation of the surface curvature only involves the second derivative of Eq. (6) with respect to {u,v}, constants *d* and *e* are zero. This reduces the bi-quadratic surface patch to

(7) $$S\left(u,v\right)=au^{2}+2buv+cv^{2}.$$

In the present work, the maximum and minimum principal curvatures at each node of the ventricular surface are used to calculate mean curvature $𝒦=1/2\left(k_{1}+k_{2}\right)$, where $k_{1}$ is the maximum principal curvature and *k_2_* is the minimum principal curvature. Figure 4 shows a graphical representation of the principal curvatures and their corresponding principal directions. $\left\{k_{1},k_{2}\right\}$ are related to the fitting constants of each surface patch and are given by

(8) $$k_{1}=a+c+\sqrt{{\left(a-c\right)}^{2}+4b^{2}}\;\;and\;k_{2}=a+c-\sqrt{{\left(a-c\right)}^{2}+4b^{2}}.$$

### 2.4 PvWMH thickness measurement

WMH thickness is defined as the distance between the ventricular wall where there is a WMH and the outer perimeter of the pvWMH mask. A custom Matlab (The Math-Works, Inc., Natick MA) code was prepared to determine the outward facing normal vector for each node along the ventricular wall that overlaps with a pvWMH location. The algorithm then identifies all elements that intersect with the normal vector and stops when those elements no longer belong to the pvWMH subgroup. An individual WMH thickness measurement is then defined as the maximum distance between the respective node on the ventricular wall and the four nodal coordinates of the furthest tetrahedral WMH-element along the normal direction. To ensure correct evaluation of WMH thicknesses, results were visually inspected and adjusted if necessary. For nodes along the ventricular wall that do no overlap with WMH locations, WMH thickness is set to zero.

### 2.5 Statistical methods

Statistical analysis was performed in Matlab. Results are reported as mean ± standard deviation. A two-sample *T* test was used to determine if ependymal cell stretch in pvWMH locations is statistically different in comparison to ependymal cell stretch in adjacent healthy white matter. To obtain an independent data set, we formed a first group that contains the nodes of all elements forming the ventricular surface that coincide with WMH locations. The second group contains the nodes of elements in proximity to WMH regions. Specifically, we search for all elements that are adjacent to a WMH region. We repeat this search a second time and identify all elements connected to the previous list. Figure 10 in the Appendix (Sect. 6.2) shows the resulting classification of the ventricular surface into WMH nodes (associated with elements in red) and adjacent healthy nodes (associated with elements in blue). Test statistic *t*, number of degrees of freedom, and *p* value are reported.

## 3 Results

### 3.1 Ventricular deformation during peak hemodynamic pressure

**Fig. 5:**
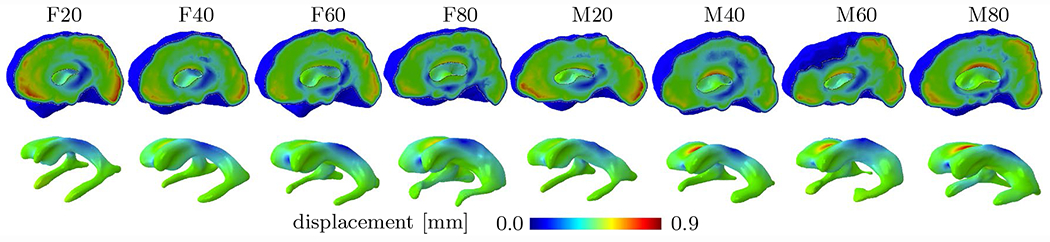
Displacement magnitude at maximum loading shown for all eight subjects. The 1st row shows a sagittal view of each full brain model while the 2nd row shows the displacement of the ventricular wall that is lined by ependymal cells. Average maximum displacement is 0.78 ± 0.27 mm (mean ± st.dev) for male subjects and 0.63 ± 0.05 mm (mean ± st.dev) for female subjects

Figure 5 shows the displacement field for all eight models. Maximum displacements differ between all models by at most 0.28 mm and consistently exhibit highest displacements in the gray matter layer in the frontal and occipital lobe, as well as the main body of the lateral ventricles. Average maximum displacement is 0.63 ± 0.05 mm (mean± st.dev) in female models with a maximum displacement of 0.57 mm, 0.65 mm, 0.62 mm, and 0.68 mm for F20, F40, F60, and F80, respectively; average maximum displacement is 0.78 ± 0.27 mm in male models (28% higher than female models) with maximum displacements of 0.68 mm, 0.82 mm, 0.75 mm, and 0.85 mm for M20, M40, M60, and M80, respectively. The lateral ventricles primarily undergo radial expansion due to the lateral ventricular pressure with the main body consistently displacing most, followed by the anterior and posterior portions of the main body’s edges. Observed displacement values compare well against existing literature data on ventricular wall deformation that observed maximum wall motion ranging from around 0.12 mm in healthy subjects [67] to about 6.5 mm in subjects with glioblastoma [68].

### 3.2 Distribution of ventricular wall loading

**Fig. 6:**
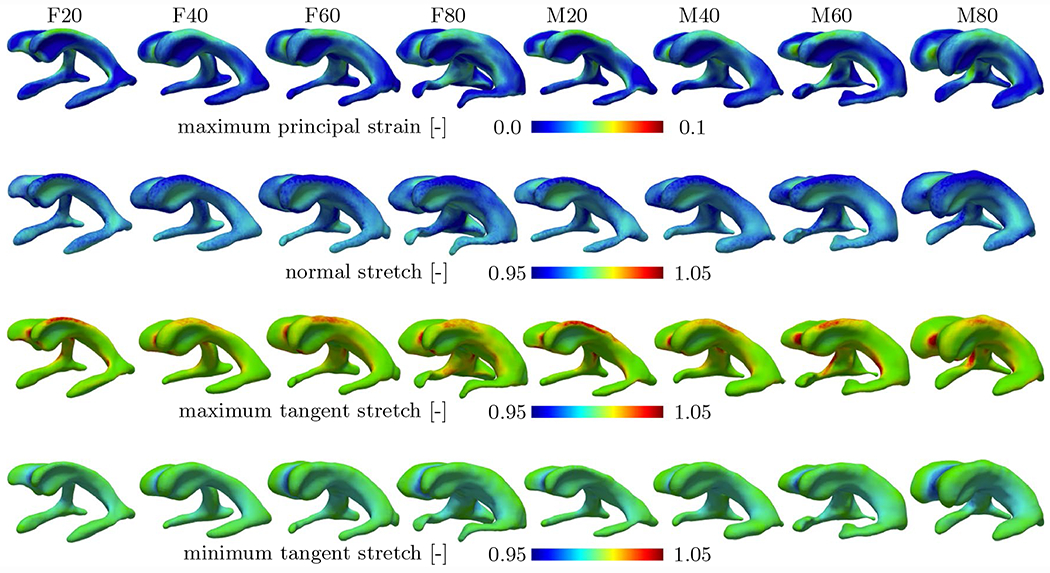
Distribution of maximum principal strain (1st row), normal stretch (2nd row), maximum tangential stretch (3rd row), and minimum tangential stretch (4th row) for all eight subjects. The peak strain for all the subjects is located around the anterior and posterior horns. Maximum ependymal cell compression (normal stretch) as well as maximum ependymal cell tension (maximum tangential stretch) are visible on the roof of the lateral ventricle’s main body as well as the edges along the main body

Figure 6 summarizes simulation output for all eight models and shows maximum principal strain, normal stretch, and maximum and minimum tangential stretch of the ventricular surface. Across all models, the edges of the ventricle’s main body as well as anterior and posterior horns are loaded most. Qualitatively, all eight subjects exhibit a similar distribution of loads irrespective of lateral ventricular volume. Nearly the entire ventricular wall undergoes compression in the normal direction with edges of the main body and the three horns being compressed most. Furthermore, maximum and minimum tangential stretches both localize in the same locations. Maximum tangential stretch is 1.053, 1.052, 1.053, and 1.062 (mean ± st.dev 1.055 ± 0.005) for female subjects F20 through F80, and 1.055, 1.067, 1.068, and 1.067 for male subjects M20 through M80 (mean ± st.dev 1.064 ± 0.006), respectively; highest maximum tangential stretch is typically observed at the anterior and posterior edges of the main body. Minimal normal stretch, i.e., maximum cell compression, is 0.924, 0.929,0 .924, and 0.935 for F20, F40, F60, and F80 (mean ± st.dev 0.928 ± 0.005); and 0.908, 0.921, 0.925, and 0.920 for M20, M40, M60, and M80 (mean ± st.dev 0.9185 ± 0.007). Comparison of stretches along the edges of the main body, however, shows that normal and maximum tangential stretches are more extreme in smaller models (F/M20 and F/M40) than in larger models (F/M60 and F/M80). That means, that larger lateral ventricles experience lower mechanical loading despite equally prescribed ventricular and subarachnoid space pressures.

### 3.3 Ventricular geometry and WMH properties

**Fig. 7:**
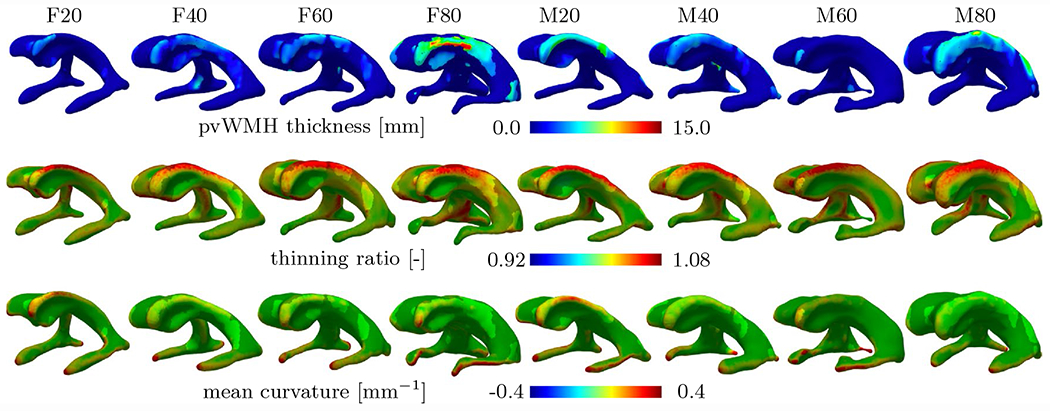
Quantification of pvWMH properties for each of the eight subjects. PvWMH thickness (1st row) was measured as the normal distance between a point on the ventricular wall to the periphery of the pvWMH area. Maximum pvWMH thickness of 18.8 mm was observed in model F80 and no clear trend with respect to ventricle size and WMH thickness was observed. Ependymal cell thinning (2nd row), i.e., the ratio between ependymal cell area stretch over apical-basal compression, is most prominent along the edges of the main body as well as in the anterior and posterior horns across all eight subjects. A maximum thinning of 1.08 is observed. Lastly, mean curvature (3rd row) across the ventricular wall is a geometric property that serves as a marker for regions of elevated lateral ventricular loading. Mean curvature is highest along the edges of the main body, in the horns, and near the anterior and posterior ends of the main body

Figure 7 shows pvWMH thickness, thinning ratio, and mean curvature of all eight models to illustrate the spatial correlation between ventricular wall loading and the location of pvWMHs. PvWMHs appear predominantly on both edges of the main body and in the anterior and posterior horns. In those same locations, ependymal cell thinning and mean curvature are higher than anywhere else along the ventricular wall. In Fig. 7, we visualize the overlap between extreme mechanical loads and pvWMH location, by highlighting surface patches exposed to a pvWMH and matting all other regions. Maximum pvWMHs thickness is 4.044 mm, 14.494 mm, 6.231 mm, and 18.747 mm for female models F20 through F80; and 11.281 mm, 18.266 mm, 4.681 mm, and 16.024 mm for male models M20 through M80. Although there is no statistically significant trend of maximum pvWMH thickness for increasing lateral ventricular volume, an overall increase in affected ventricular surface is observed. Specifically, ventricular surface area covered by a pvWMH gradually increases with lateral ventricular volume with 659.18 mm^2^ for F20, 2233 mm^2^ for F40, 1885 mm^2^ for F60, and 3600 mm^2^ for F80; and 1502 mm^2^ for M20, 2827 mm^2^ for M40, 374.54 mm^2^ for M60, and 3651 mm^2^ for M80. Ependymal cells undergo significant thinning in locations along the edges of the lateral ventricles’ main body that are covered by pvWMHs, see highlighted sections in the ventricles in second row of Fig. 7. Maximum thinning ratios are 1.15, 1.13, 1.14, and 1.11 for female models F20 through F80 (mean ± st.dev 1.13 ± 0.017); and 1.2, 1.15, 1.14, and 1.16 for male models M20 through M80 (mean ± st.dev 1.16 ± 0.032). Mean curvature, shown in the third row of Fig. 7, is highest along the edges of the lateral ventricles’ main body, at the anterior and posterior end of the main body, and around all three horns. Maximum mean curvature is 0.8 mm^−1^, 0.78 mm^−1^, 0.63 mm^−1^ , and 1.0 mm^−1^ for female models (mean ± st.dev 0.8 ± 0.15 mm^−1^); and 0.95 mm^−1^, 0.95 mm^−1^, 1.37 mm^−1^ , and 0.64 mm^−1^ for male models (mean ± st.dev 1.09 ± 0.245 mm^−1^). Given the small sample size in the present work, no explicit sex-specific differences can be observed. We observed, however, that ependymal cell stretches and the thinning ratio are very similar for our female and male models. Irrespective of gender, the smallest ventricles, i.e., F/M20 and F/M40, exhibit higher mean curvatures along the edges of the main body in comparison to larger models, i.e., F/M60 and F/M80. This is key to the hypothesis that mechanical loading of ependymal cells is one of the most important factors in pvWMH formation and the early growth phase. Principal curvatures *k*_1_ and *k*_2_ are provided in Fig. 9 in the Appendix. Inferior horns exhibit very high mean curvature values without corresponding pvWMHs. In general, literature generally does not discuss WMH formation near the inferior horns of the lateral ventricles. At the same time, it is important to note that the FLAIR sequences used on our subjects focus on the ventricle’s main body and do not provide good representation of the temporal horns. This is a limitation of most commonly used FLAIR sequences and needs to be addressed in future work. In summary, it appears that elevated mean curvature and high thinning ratio must coincide to create a mechanical loading state that exceeds the physiological limit of ependymal cells to prevent CSF leakage.

### 3.4 Sensitivity analysis

**Fig. 8:**
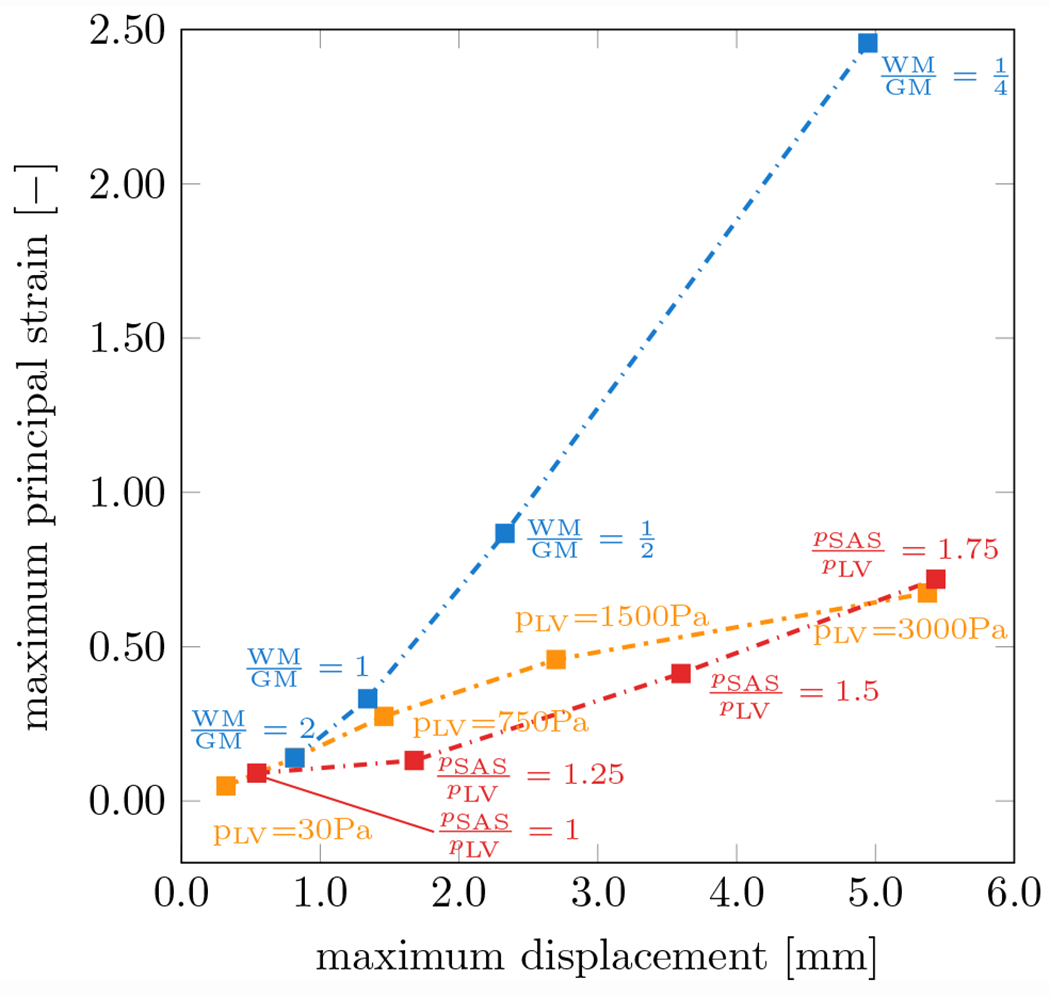
Sensitivity analysis of the M40 FE model with respect to material properties, maximum ventricular loading, and pressure ratio between lateral ventricle and subarachnoid space. Maximum principal strain and maximum displacement are determined for lateral ventricular pressures ranging from 30 to 3000 Pa, pressure ratios *p*_SAS_/*p*_LV_ ranging from 1 to 1.75, and white-to-gray matter stiffness ratios ranging from 1/4 to 2

**Fig. 9:**
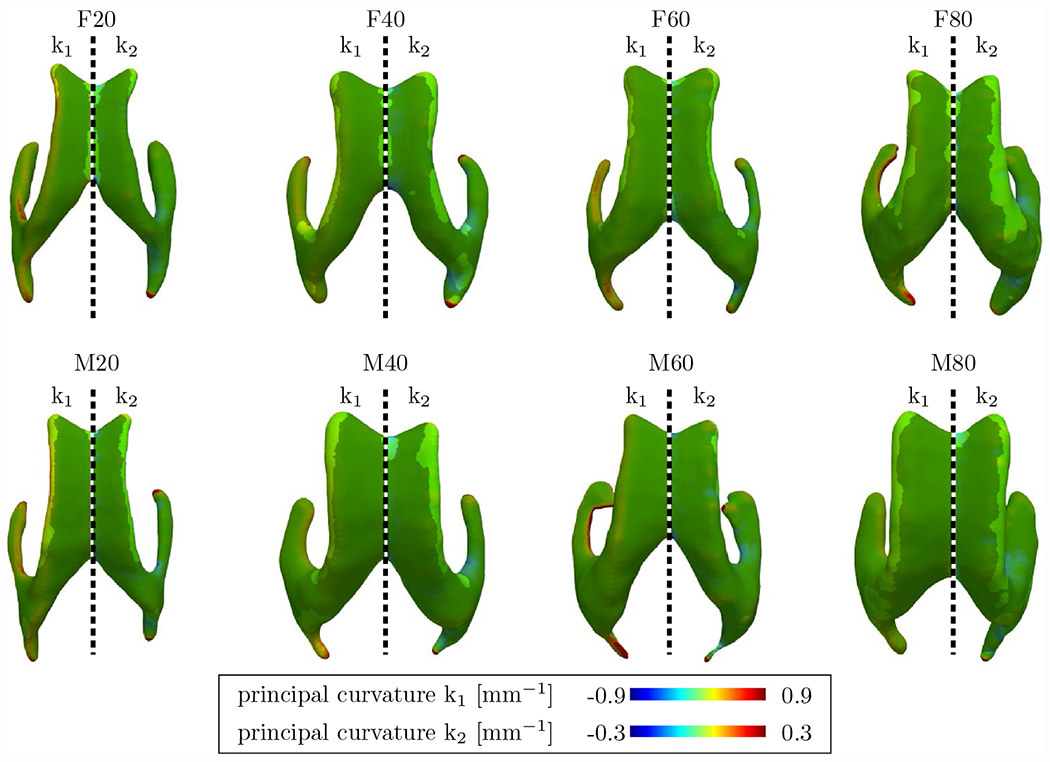
Principal curvatures *k*_1_, shown on the left, and *k*_2_, shown on the right, for all eight models. *k*_1_ is up to three times higher than *k*_2_ and is highest along the edges of the main body as well as all three horns

Figure 8 shows sensitivity with respect to material properties, maximum ventricular loading, and pressure ratio between lateral ventricle and subarachnoid space for the example of model M40. Maximum principal strain and maximum displacement of the ventricular wall are reported since they are representative measures for the lateral ventricle’s overall loading state. For one, maximum ventricular pressure (*p*_LV_ ) was varied from 30 to 3000 Pa to assess a wide range of intracranial pressures, see orange points. A 100-fold increase in lateral ventricular pressure over the reference pressure used throughout the present work increases maximum principal strain by a factor of 6.6 and maximum displacement by a factor of 4.8. For the other, the ratio between lateral ventricular and subarachnoid pressure was varied from 1 to 1.75 to assess the impact of the peripheral pressure on the parenchyma, see red points. Nearly doubling the subarachnoid space pressure with respect to the reference lateral ventricular pressure produced similar maximum principal strain and maximum displacement values as the first analysis with a factor of 6.7 and 5.1, respectively. Lastly, the white-to-gray matter stiffness ratio was varied from 2 to 1/4. This produced the most significant changes with respect to maximum principal strain and maximum displacements with a factor of 17.5 and 6.1, respectively.

The model’s sensitivities suggest that subjects with increased intracranial pressure are at increased risk of developing ventricular wall damage [15, 69, 70]. Moreover, even though the model shows a pronounced response to changes of the white-to-gray matter stiffness ratio, relative tissue softening on the order of 50% has not been reported in literature and is considered highly unlikely.

### 3.5 Spatial correlation between pvWMH location and mechanomarkers

Table 1 shows the results of a statistical analysis to assess the spatial overlap between mechanomarkers and pvWMH locations. Maximum principal strain, thinning ratio, and mean curvature all show statistical significance with respect to increased mean values in regions where there are pvWMHs in comparison to adjacent regions of the ventricular wall without pvWMHs (*p* < 0.001, with two exceptions: no significant differences were observed for mean curvature of model F60 and the maximum principal strain for model F80). This suggests that elevated mechanical loading associated with a high thinning ratio and high curvature colocalizes with pvWMH locations and may play a significant role in the consistent onset locations of periventricular white matter lesions. In regions with pvWMHs in comparison to the surrounding lateral ventricular wall, maximum principal strain is on average 14% higher, thinning ratio is on average 8% higher, and curvature is on average 24% higher.

## 4 Discussion

The association of pvWMHs with cognitive impairment, depression, decreased mobility, and increased risk of stroke provides ample motivation to investigate the concise etiology of white matter lesion initiation and growth. The observation of pvWMHs in nearly every aged brain further stresses the need to identify possible mitigation strategies that delay pvWMH formation with the goal to delay cognitive decline and reduce the risk of stroke.

### 4.1 Risk factors for pvWMH formation

Thus far, pvWMHs are associated with vascular risk factors such as diabetes, smoking, and hypertension, and are, therefore, considered to be another manifestation of cerebral small vessel disease. Cerebral small vessel disease generally refers to various pathophysiological processes associated with small arteries, arterioles, capillaries, and small veins, and includes vessel stiffening, lacunes, microbleeds, superficial siderosis, perivascular spaces, microinfarcts, and blood–brain barrier dysfunction [71]. The vascular origin of pvWMHs is typically motivated by imaging data that shows significant spatial overlap between altered diffusion tensor imaging parameters such as increased mean diffusivity and reduced fractional anisotropy, and bright-appearing spots in FLAIR images [17, 19, 32]. Increased mean diffusivity indicates elevated diffusion of water molecules and declining fractional anisotropy is a sign of more pronounced isotropic diffusion of water mobility. Both are the result of white matter degeneration in the form of progressive demyelination, axonal damage, microglial activation, and neuroinflammation [72, 73]. FLAIR imaging is designed to dampen signal from CSF while the brain appears similar to T2-weighted images with gray matter brighter than white matter; simultaneously, fluid trapped in the parenchyma appears bright white, and therefore, provides a very high WMH-tissue contrast that allows to delineate WMHs [74, 75]. The integration of FLAIR and diffusion tensor imaging has shown, that normal appearing white matter undergoes microstructural changes prior to transitioning into WMHs [19]. Changes in water composition and content in the vicinity of pvWMHs pre-date microstructural changes such as white matter lesions. The consequence of age-related and hypertension-related cerebral small vessel disease on the brain is mainly associated with vessel wall rupture leading to anything from microbleeds to hemorrhaging, chronic white matter hypoperfusion causing the degeneration of myelinated axons due to selective oligodendrocyte death, and acute occlusion of small vessels associated with lacunar infarcts [2]. Ischemic forms of cerebral small vessel disease are caused by blood–brain barrier damage resulting in extravascular plasma proteins, such as fibrinogen and albumin, which disrupt transmembrane ionic gradients and transport pathways and facilitate local inflammation and oligodendrocyte apoptosis [76]. Accelerating age-related, noninflammatory collagen deposition results in walls thickening and causes severe periventricular venous stenosis and vessel stiffening [77–79]. These vascular changes lead to increased pulsatility, restricted vasodilation, and impaired normal perivascular fluid flushing and removal of waste [80]. Limited cerebral vasoreactivity and lower cerebral pulsatility gradually creates a chronic state of white matter hypoperfusion [81].

From a mechanics perspective, the above mentioned pathological mechanisms have one important common outcome: they reduce local white matter tissue stiffness. Tissue softening, as shown in the sensitivity analysis summarized in Fig. 8, has the strongest impact on lateral ventricular wall deformations and ependymal cell stretch. Gradual deterioration of white matter integrity, i.e., demyelination, neuro-inflammation, blood–brain barrier disruption, and gliosis, leads to softer white matter tissue [56, 82, 83]. The resulting increase in ependymal cell loading raises the probability of CSF leakage into white matter further exacerbating pvWMH growth. Moreover, softer white matter leads to increased compression of the parenchyma during hemodynamic loading and contributes to vessel collapse and tissue hypoperfusion. As such, existing risk factors for pvWMH formation inherently have a mechanistic component that is associated with excessive mechanical loading of the ventricular wall causing membrane dysfunction and breakdown of transependymal bulk ow. This mechanistic view of pvWMH progression is supported by pathology studies that report discontinuous ependyma, gliosis, and repair of ependymal cell loss along the ventricular wall by astrogliosis [38, 39, 42, 43]. Descriptions of ventricular wall integrity range from intact ependymal cell coverage, to partial loss of ependymal cells associated with surface astrocytic processes, and ultimately dense areas of surface gliosis [39].

### 4.2 Ventricular shape changes

Ventricular enlargement is a hallmark feature of the aging brain and results from gradual white and gray matter atrophy [60, 84]. Age-related ventricular volume growth is characterized by a mostly uniform expansion of the lateral ventricle [39, 85]. Longitudinal imaging studies have shown that the main body as well as anterior, posterior, and inferior horns expand isotropically rather than undergoing localized bulging [84]. This is important for the present work, as this implies that the distribution of wall loading does not change significantly with respect to ventricular volume. In other words, anterior and posterior horns, as well as the lateral edges of the main body will consistently be loaded most and continuously contribute to ependymal cell deterioration. Based on cross-sectional data, Fjell et al. observed a 4.40% volume increase of the lateral ventricle over a 1-year period in 142 healthy elderly participants between 60 and 91 years of age [86]. Coupe et al. reported a near sixfold increase of lateral ventricles over a 80-year period [87]. Other work has shown that denudation of the ventricular wall can also be a driver for ventricular expansion [39]. The present work covers a broad range of ventricular geometries that are representative of ventricular enlargement observed across a large age-span and can be considered further validation of the posed hypothesis that mechanical loading of the ventricular wall is a critical factor for the emergence of WMHs. Future work should incorporate atrophy as a driving force behind ventricular loading coupled with the emergence and growth of periventricular white matter lesions. For the example of model M40, cerebral atrophy was simulated by uniformly shrinking white matter and gray matter tissue until lateral ventricular volume increased by 20%. The resulting loading of the lateral ventricular wall reached 0.08 maximum principal strain, a maximum tangential stretch of 1.14, and a maximum normal stretch of 1.04. This marks a drastic increase in comparison to the loads observed in the present work and clearly indicates that cerebral atrophy leads to significant mechanical loading of the ventricular wall. Figure 11 in the Appendix shows correlations between CSF volume and pvWMH-related properties including, but not limited to, lateral ventricular volume, total pvWMH volume, pvWMH surface area covering the ventricular wall, Fazekas score, and mechanomarkers of ependymal cell loading. CSF volume is statistically significant correlated (*p* < 0.05) with lateral ventricular volume, pvWMH volume, and pvWMH surface area covering the ventricular wall. Subject age and Fazekas score are weakly correlated with CSF volume. No statistically significant sex differences are observed. While subject data shows pronounced variations with respect to fluid and pvWMH volumes (Fig. 11B–D), mechanomarkers are much more uniformly distributed across the subject’s varying CSF volumes (Fig. 11F–H).

### 4.3 Limitations

The present work is not without limitations. First, the current study is based on eight subjects due to the effort required to generate anatomically accurate finite-element models. This small sample size limits the ability to derive statistically significant trends of CSF and anatomically accurate finite-element models volumes and pvWMH progression. That being said, each model demonstrates significant spatial overlap with respect to mechanomarkers and the presence of pvWMHs. Mechanically, peak ependymal cell stretch, thinning ratio, and maximum principal strain will always appear in locations with maximum mean curvature. Secondly, locations with elevated mechanomarkers do not always coincide with pvWMH locations for all models equally. This shows that mechanical loading of the ventricular wall is likely not the only factor causing pvWMH formation. The selected subjects do show, however, that pvWMHs consistently form in the horns where there are highest loads first. Lastly, the present work does not address age- and damage-related brain property changes and the fact that pvWMHs grow over time. Future work should address the impact of white matter degeneration and the progressive deterioration of pvWMHs from thin linings to caps to diffuse WMHs in deep white matter regions. The corresponding damage model should establish a relationship between atrophy-driven ventricular enlargement, white matter stiffness changes, and white matter lesion growth due to a leaking CSF-parenchyma barrier.

## 5 Conclusions

PvWMHs are a common observation in the aging brain and are associated with cognitive decline due to white matter degeneration. The underlying mechanisms causing lesion formation and subsequent growth remain understudied and require a unifying theory to rationalize differences between periventricular, deep, and subcortical WMHs. Here, eight subject-specific finite-element models consistently demonstrated that peak mechanical loading of the ventricular wall spatially overlaps with pvWMH locations for a broad spectrum of ventricular shapes and volumes. Specifically, expansion of the ventricular wall causes ependymal cells to be stretched thin, creating significant tension on cadherin junctions that form a semi-permeable membrane between parenchyma and CSF. Simulation results suggest that ependymal cell thinning is a particularly sensitive mechanomarker for likely pvWMH locations. This implies that mechanical loading of the ventricular wall, in combination with vascular contributions in periventricular white matter, plays a critical role in pvWMH pathology. The present findings could lead to the earlier identification of subjects at risk of developing advanced pvWMHs and to the identification of possible pharmacological interventions that mitigate periventricular white matter deterioration in the aging brain.

## Figures and Tables

**Table 1:** Statistical analysis to correlate pvWMH location and proposed mechanomarkers

Model	Maximum principal strain		Thinning ratio		Mean curvature	
	WMH/no WMH	*T* test	WMH/no WMH	*T* test	WMH/no WMH	*T* test
F20	0.015 ± 0.017	*t*(2403) = 4.559	1.030 ± 0.027	*t*(2403) = 4.239	0.125 ± 0.095	*t*(2403) = 6.851
	0.012 ± 0.016	*p* < 0.001	1.025 ± 0.027	*p* < 0.001	0.100 ± 0.074	*p* < 0.001
F40	0.014 ± 0.010	*t*(6919) = 5.147	1.035 ± 0.021	*t*(6919) = 5.683	0.091 ± 0.063	*t*(6919) = 3.652
	0.012 ± 0.011	*p* < 0.001	1.032 ± 0.023	*p* < 0.001	0.085 ± 0.066	*p* < 0.001
F60	0.020 ± 0.016	*t*(5781) = 9.489	1.045 ± 0.025	*t*(5781) = 9.126	0.081 ± 0.048	*t*(5781) = 0.835
	0.016 ± 0.013	*p* < 0.001	1.038 ± 0.027	*p* < 0.001	0.082 ± 0.076	*p* = 0.404
F80	0.013 ± 0.012	*t*(10038) = 1.228	1.037 ± 0.023	*t*(10038) = 7.578	0.082 ± 0.080	*t*(10038) = 4.786
	0.013 ± 0.013	*p* = 0.220	1.034 ± 0.024	*p* < 0.001	0.074 ± 0.069	*p* < 0.001
M20	0.021 ± 0.019	*t*(4729) = 14.888	1.047 ± 0.030	*t*(4729) = 13.512	0.151 ± 0.093	*t*(4729) = 19.692
	0.013 ± 0.014	*p* < 0.001	1.035 ± 0.027	*p* < 0.001	0.097 ± 0.089	*p* < 0.001
M40	0.017 ± 0.016	*t*(8176) = 10.704	1.043 ± 0.023	*t*(8176) = 11.109	0.116 ± 0.097	*t*(8176) = 24.365
	0.013 ± 0.010	*p* < 0.001	1.038 ± 0.020	*p* < 0.001	0.066 ± 0.058	*p* < 0.001
M60	0.019 ± 0.012	*t*(1679) = 8.312	1.048 ± 0.020	*t*(1679) = 6.093	0.187 ± 0.183	*t*(1679) = 15.380
	0.014 ± 0.013	*p* < 0.001	1.042 ± 0.022	*p* < 0.001	0.081 ± 0.086	*p* < 0.001
M80	0.018 ± 0.013	*t*(9215) = 9.671	1.047 ± 0.020	*t*(9215) = 16.435	0.078 ± 0.056	*t*(9215) = 12.803
	0.015 ± 0.013	*p* < 0.001	1.039 ± 0.023	*p* < 0.001	0.061 ± 0.055	*p* < 0.001

**Fig. 10:**
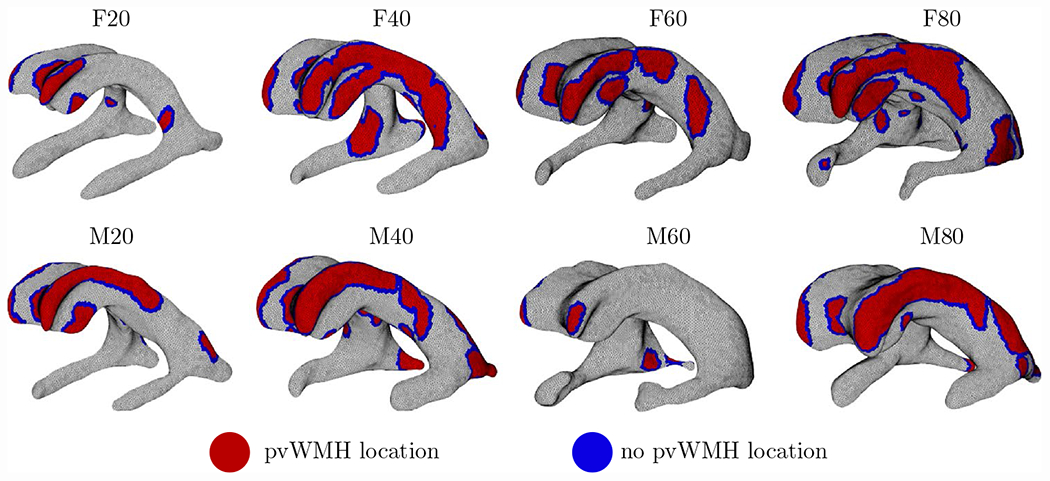
Separation of nodes into a group where there are pvWMHs (red) and neighboring healthy tissue (blue). These two groups were used to determine statistical significance between mechanomarkers and pvWMH location (color figure online)

**Fig. 11:**
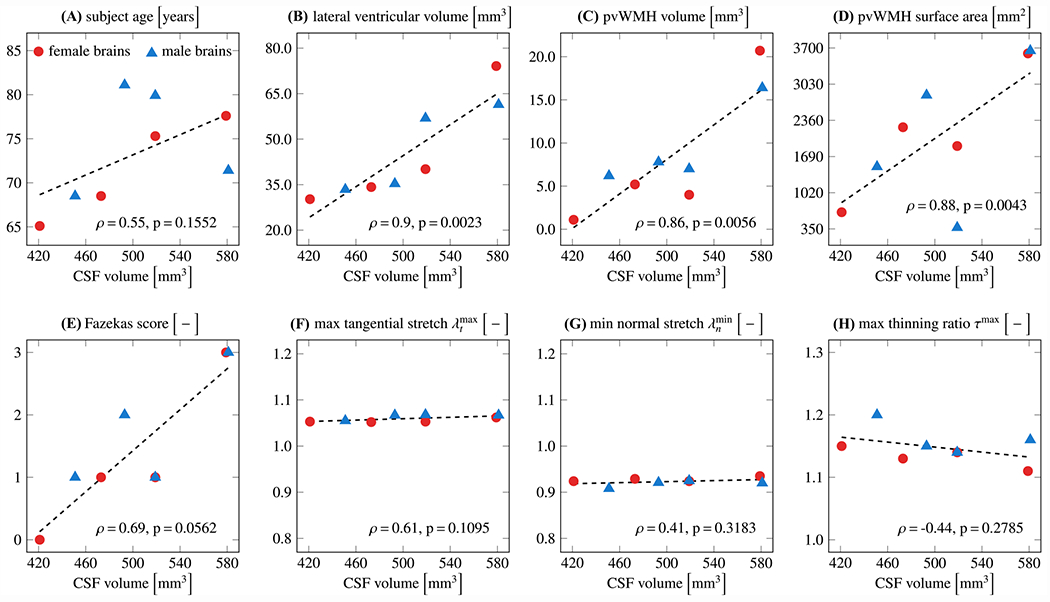
Correlation between CSF volume and **A** subject age, **B** lateral ventricular volume, **C** pvWMH volume, **D** pvWMH area covering the ventricular surface, **E** Fazekas score, **F** maximum tangential stretch on the ventricular surface, **G** minimum normal stretch on the ventricular surface, and **H** maximum thinning ratio on the ventricular surface. Female subjects are marked by red points, male subjects are marked by blue triangles. Trend lines are plotted based on a linear regression model and Pearson’s correlation factor *ρ* and the corresponding *p* value are reported

Mean ± st.dev of maximum principal strain, thinning ratio, and mean curvature are reported for regions with pvWMHs and neighboring lateral ventricular wall sections for all eight models, see Fig. 10 in the Appendix. The two-sample *T* test results are reported as test statistic, degrees of freedom, and significance level

## Appendix

### Principal curvatures of the lateral ventricles

Figure 9 shows the principal curvatures for all eight models. Left shows the first principal curvature and the right image shows the second principal curvature. There is extensive symmetry in the models with respect to left and right parts of the lateral ventricles.

### Groups formed for statistical analysis

The statistical analysis performed to determine significance between mechanomarkers and pvWMH locations is based on the two groups highlighted in Fig. 10. Red surface patches, and their respective nodes, belong to the group where there are pvWMHs. Their locations were determine by registering the FLAIR mask to the FE mesh of the lateral ventricles. The blue patches, and their respective nodes, were selected based on their immediate proximity to the red patches. As outlined in Sect. 2.5, two element layers around pvWMH locations were chosen in the current analysis.

### Correlation between CSF volume, subject data, and ependymal cell loading

Correlation between CSF volume and subject data and model properties are reported in Fig. 11. It is observed that increasing CSF volume is associated with an increase in age, lateral ventricular volume, pvWMH volume, and Fazekas score. Mechanical loading of ependymal cells remains fairly uniform irrespective of CSF volume. No significant sex differences are observed.
